# SARS-CoV2 vaccination status among psychiatry inpatients: a retrospective cohort analysis

**DOI:** 10.1192/j.eurpsy.2023.869

**Published:** 2023-07-19

**Authors:** H. Andreu, I. Ochandiano, L. Olivier, Ò. De Juan, L. Bueno, E. Cesari, J. I. Mena, S. Salmerón, P. Barrio

**Affiliations:** 1 Psychiatry and Psychology Department; 2 Addictive Behaviors Unit, Clinical Neuroscience Institute, Hospital Clínic, Hospital Clínic de Barcelona, Barcelona, Spain

## Abstract

**Introduction:**

The coronavirus infectious disease 2019 (COVID-19) pandemic has had a deleterious impact in many areas. Given this, efforts have focused on developing effective vaccines and vaccination campaigns have been carried out prioritizing population at risk. This should include mental health patients since they are at higher risk of developing complications or ending up in a critical status. Since it may be sometimes difficult for these patients to access vaccination, hospitalization may be a window of opportunity to evaluate and offer vaccination.

**Objectives:**

This study aims to retrospectively assess vaccination status and offer during admission of psychiatry inpatients at Hospital Clínic of Barcelona during a 6-month period, in order to determine if there are differences regarding vaccination rates compared to general population and between main diagnostic categories.

**Methods:**

We retrospectively evaluated all admitted patients to the acute psychiatry ward. The main collected variables included age, gender, main psychiatric diagnosis, presence of organic comorbidities, vaccination status at admission and vaccination offer during admission. We used descriptive statistics to extract most of the information. A binary logistic regression was also conducted to evaluate whether the main diagnosis, age and gender had some influence upon vaccination status at admission.

**Results:**

Between January 1^st^ and June 30^th^ of 2022, 216 patients were admitted to the psychiatry ward. A total of 42% were female, with a mean age for the whole sample of 42.8 years (SD 14.7). More than half were current smokers (55%), and 46% of the patients had at least one significant organic comorbidity. The percentages of main diagnosis were as follows: addiction 21.3%; bipolar disorder 18.5%; schizophrenia 18.1%; non-specified psychosis 14.4%; depression 7.4%; cognitive impairment 0.9%; personality disorders 6.9%.

Vaccination status was available for 187 patients (86.6%). Of these, 78 patients were fully vaccinated, 68 had an incomplete vaccination status and 41 patients had not received any dose. No differences on the vaccination status were seen based on the psychiatric diagnosis. Among patients with incomplete or no vaccination, 19 patients (17.4%) were offered a vaccination dose. A total of 11 patients accepted and received it (57.9%). In the logistic regression model, the only significant variable predicting an increase in the likelihood of being fully vaccinated was age, with every year of age increasing the probability of full vaccination by 6%.

**Conclusions:**

Our data suggest that routine screening of vaccination status during psychiatric admission and improved strategies for vaccination offer and acceptance should become a priority in psychiatric wards. Given the impact of the pandemics, and the likelihood of new waves or even new pandemics, more research on vaccination strategies among mental health patients is warranted.

**Disclosure of Interest:**

None Declared

